# Exploring the expectations, experiences and tensions of refugee patients and general practitioners in the quality of care in general practice

**DOI:** 10.1111/hex.13411

**Published:** 2021-12-23

**Authors:** Pinika Patel, Danielle M. Muscat, Lyndal Trevena, Dipti Zachariah, Hanaa Nosir, Nishanthie Jesurasa, Amina Hadi, Sarah Bernays

**Affiliations:** ^1^ Sydney School of Public Health, Faculty of Medicine and Health The University of Sydney Sydney New South Wales Australia; ^2^ Ask Share Know: Rapid Evidence for General Practice Decisions (ASK‐GP), Centre for Research Excellence, School of Public Health, Faculty of Medicine and Health The University of Sydney Sydney New South Wales Australia; ^3^ Sydney Health Literacy Lab, School of Public Health, Faculty of Medicine and Health The University of Sydney Sydney New South Wales Australia; ^4^ Multicultural Health, Integrated and Community Health Directorate Western Sydney Local Health District Cumberland New South Wales Australia; ^5^ Department of Global Health and Development, Faculty of Public Health and Policy London School of Hygiene and Tropical Medicine London UK

**Keywords:** communication, patient engagement, refugee

## Abstract

**Background:**

Refugees and asylum seekers arrive in the Australian community with complex health needs and expectations of healthcare systems formed from elsewhere. Navigating the primary healthcare system can be challenging with communication and language barriers. In multicultural societies, this obstacle may be removed by accessing language‐concordant care. Emerging evidence suggests language‐concordance is associated with more positive reports of patient experience. Whether this is true for refugees and asylum seekers and their expectation of markers of quality patient‐centred care (PCC) remains to be explored. This study aimed to explore the expectations around the markers of PCC and the impacts of having language‐concordant care in Australian primary healthcare.

**Methods:**

We conducted semi‐structured individual in‐language (Arabic, Dari, and Tamil) remote interviews with 22 refugee and asylum seekers and 9 general practitioners (GPs). Interview transcripts were coded inductively and deductively, based on the research questions, using Thematic Analysis. Extensive debriefing and discussion took place within the research team throughout data collection and analysis.

**Results:**

Community member expectations of markers of PCC are constantly evolving and adapting based on invisible and visible actions during clinical encounters. Challenges can occur in the clinical encounter when expectations are ‘unsaid’ or unarticulated by both community members and GPs due to the assumption of shared understanding with language concordant care. Expectations of what constitutes satisfactory, quality PCC are dynamic outcomes, which are influenced by prior and current experiences of healthcare.

**Conclusion:**

This study highlights the importance of understanding that language concordant care does not always support aligned expectations of the markers of quality PCC between community members and their GP. We recommend that GPs encourage community members to provide explicit descriptions about how their prior experiences have framed their expectations of what characterizes quality PCC. In addition, GPs could develop a collaborative approach, in which they explain their own decision‐making processes in providing PCC to refugees and asylum seekers.

**Patient or Public Contribution:**

Bilingual researchers from multicultural backgrounds and experience working with people from refugee backgrounds were consulted on study design and analysis. This study included individuals with lived experiences as refugees and asylum seekers and clinicians as participants.

## 1 INTRODUCTION

In multicultural societies, such as Australia, healthcare providers are increasingly providing care to patients from various ethnic backgrounds, including people arriving on humanitarian grounds as refugees and asylum seekers.^1^ Refugees are defined as those who have been granted permanent residency and asylum seekers are defined as whose claim for refugee status has not yet been determined. Both are individuals who have fled their country of origin due to persecution, conflict, violence and human rights violations.^2^ Many refugees and asylum seekers arrive in their resettlement countries with complex psychological and physiological needs and with expectations of healthcare systems formed from elsewhere.^3^ Numerous factors affect how refugees and asylum seekers navigate primary healthcare services, which are often the first point of care for these individuals in Australia.^4^, ^5^, ^6^, ^7^ These include lack of familiarity with the Australian health system, language barriers, mistrust, anxiety and financial constraints. Healthcare providers report being challenged by time constraints, a lack of familiarity with refugee health issues, language barriers and interpreter use.^4^, ^5^, ^8^, ^9^

Communication is paramount in building confidence and trust between the healthcare provider and patients from culturally and linguistically diverse backgrounds.^10^ How this is experienced is shaped in part by patients' cultural views, language proficiency and patient's perceptions of the quality of care.^11^ To support the accurate and accessible translation of key messages within the clinical consultation professional interpreters have been recommended to improve patient health outcomes.^12^, ^13^ However, even interpreters when taken up concerns have been reported about availability, access, confidentiality, and the accuracy of translation.^5^, ^14^

Some of these concerns with professional interpreter use could be negated through language concordant care between the healthcare provider and patient. Language concordant care is defined as situations where both the patient and healthcare provider speak to each other in their shared language.^15^ The evidence suggests that language concordant care may also serve as a potential mechanism to enhance patient trust, which could contribute to improved health outcomes, including reported patient satisfaction and quality of interpersonal care,^16^, ^17^ and advancing health equity.^18^, ^19^

While language‐concordant care provides opportunities to improve communication and trust,^20^ it is still unclear whether it results in being able to address the individual's expectations of quality care. Patient‐centred care (PCC) is a principle applied in healthcare aiming to enhance interpersonal interaction, by placing considerable emphasis on tailoring care to meet the individual's needs and psychosocial circumstances.^21^, ^22^ Providing PCC in a multicultural society calls for additional considerations when practicing PCC with someone from a culture different from one's own, where there may be a divergence between patients and providers' experiences and understandings of social norms, values and communication.^23^ Key to achieving PCC in these circumstances is adapting communication approaches to ensure that the patient is engaged in being able to understand and participate in a meaningful and constructive dialogue in their clinical care.^24^ Language concordant care, in providing an opportunity to address and remove communication barriers, may be critical in supporting the provision of PCC to help care for refugees and asylum seekers at an individual level.

Existing literature on the impact of language concordant care on the delivery of PCC for refugee and asylum seekers, and how this in turn influences their expectations and reported quality of care, is limited. How the varying understandings of the cultural norms, race/ethnicity, migrant visa status, country of origin and time in the current country may influence patients' expectations and understanding of their clinical care has also received little attention.^25^ Providing PCC to refugees and asylum seekers requires tailoring care to the individual with consideration of their communication and language needs, as well as their likely lack of familiarity with the Australian delivery of primary healthcare.^8^

In this paper, we focus on giving a voice to community members from refugee and asylum seeker backgrounds, and their expectations of quality care and how that impacts their overall communication with their general practitioner (GP). The aim of this study is to provide insight into refugee and asylum seekers' communication experiences and expectations with their GP in more detail, where individuals seek out GPs who speak the same language as them. With this qualitative study, we focused on two main research questions: (1) How do expectations of markers of PCC impact the GP interaction? (2) What is the influence of language concordance care on their lived experience of primary healthcare?

## 2 METHODS

### 2.1 Study design

Adopting a qualitative methodology, using semi‐structured interviews, this study sought to describe the experiences of people from refugee and asylum seeker backgrounds receiving care from their GPs, and discuss the barriers and facilitators to their communication and interaction during consultations. In response to the COVID‐19 pandemic and social distancing measures and requirements, the study was designed to allow for remote recruitment and data collection to ensure the safety of the community members and research team. The reporting of this qualitative study follows the consolidated criteria for reporting qualitative research (COREQ criteria). Ethical approval was obtained through the Western Sydney Local Health District Ethics Office (ref: 2020/ETH00246).

### 2.2 Setting

The study was conducted in Western Sydney, a local health district in Sydney, Australia. This district was selected due to the large culturally and linguistically diverse population; more than 50% of people in the Western Sydney Local Health District (WSLHD) speak a language other than English at home.^26^ We selected participants from some of the most numerically significant language groups being served in the local health district: Arabic, Dari and Tamil. In addition to being three of the top five languages spoken by people from refugee and asylum seeker backgrounds in the district, these languages were purposefully chosen to reflect diversity in the drivers of migration, with variation in the region of origin and political histories.

The study was conducted in partnership with the Western Sydney multicultural health team (D. Z., H. N., N. J., A. H.), who were involved in the study design, recruitment, data collection, data management and analysis. As part of our participatory approach, bilingual educators (H. N., N. J., A. H.) were trained to recruit and conduct qualitative interviews with the community members, given they are considered supportive, trusted partners within these communities. They were integral to each stage of the research process, including through the analysis, and write‐up.

### 2.3 Study participants

Adult community members who had arrived in Australia as refugees or asylum seekers from these three language communities, living in the WSLHD and active participants in a range of community groups, were invited to participate. Rather than imposing a threshold on the number of years since migration the inclusion criteria for this study was intentionally broad to allow for diverse experiences of community members who self‐identified as refugees. In this study, refugees are defined as those who have been granted permanent residency in Australia; these individuals have access to Medicare (bulk‐billed healthcare).^27^ The dynamic status and conditions of asylum seekers' visas mean that they have more precarious access to healthcare and Medicare.^27^, ^28^ We refer to refugee and asylum seeker community participants as community participants.

GPs who work in WSLHD were invited to participate if they had had a consultation with at least one patient from a refugee or asylum seeker background in the last year. We purposefully sampled bilingual GPs, in particular those who spoke the same languages as the community members (i.e., Arabic, Dari, Tamil).

### 2.4 Participant recruitment

Refugee and asylum seekers attending five pre‐existing community groups, including language study and parent support groups were invited to participate.

All adult community members of these groups were sent study information via video link through existing community group emailing lists and WhatsApp groups. The study videos were available in each of the three languages (Arabic, Dari, Tamil) plus English, and included basic project information and eligibility criteria. The inclusion of in‐language video invitations allowed for those with limited reading and writing skills to be able to listen to the information in their preferred language and then talk directly to someone who also spoke their preferred language. This inclusive approach to recruitment was designed to encourage the involvement of those who would perhaps have the greatest challenges in accessing and utilizing Australian healthcare and may ordinarily be indirectly excluded from research due to language. The videos were embedded into a secure online survey platform (REDCap),^29^, ^30^ and if they wished to participate community members were invited to leave their contact details.

Anyone who expressed interest was contacted by one of the bilingual educators (H. N., N. J., A. H.) in a follow‐up phone call to verbally check eligibility, discuss participation, arrange informed consent documentation (in the individual's preferred language) and set up a date and time for the interview. All community participants were given the option of being interviewed by phone or on a video call and all chose to have telephone interviews.

Community participants had three options for giving consent. The first was paper written consent, dropped off at the community centre. The second was sending a digital copy of the written consent through WhatsApp/Email. The third was providing verbal consent, in which a standardized verbal consent script, covering the key components of the participant information sheet and consent form, was read in‐language at the start of interview and audio‐recorded. Half the community participants chose option 1, returning written consent to their community centre, while the other half gave verbal consent.

Eligible GPs received an invitation flyer, containing details on how to receive further information on the aims and procedure of the study, sent via professional networks and email lists. For those who expressed interest, the first author (P. P.), contacted them to check eligibility and organize a date and time for an interview either through Zoom videoconferencing or via the telephone. All GP participants were given a choice between written and verbal consent processes; five GPs provided verbal consent and four GPs opted to return written consent. Two interviews were conducted over the phone and the other seven were conducted over Zoom videoconferencing.

### 2.5 Data collection procedure

The community member telephone interviews were conducted between February and April 2021 by the trained female bilingual educators (H. N., A. H., N. J.), each of whom was individually fluent in one of the study languages: Arabic, Dari or Tamil. All community participants who completed the interviews received a 25AUD shopping voucher, in line with local research practices.^31^ The community participant interviews on average lasted 20 min. The bilingual educators ensured the interviews were conducted at a time and place, which was convenient to the participant. All community member interviews were audio‐recorded and independent translators translated audio‐recordings and transcribed them verbatim in English.

The first author, a female public health researcher with experience in qualitative interviews, conducted all the semi‐structured GP interviews at a time convenient to the GP. These interviews were conducted between February and August 2021. All GP interview transcripts were audio‐recorded and transcribed verbatim by PP. The GP interviews lasted 30 min on average.

The community member topic guides covered the interpersonal interaction between the patient and GP, their lived experiences of care and their various communication needs during GP consultations. The GP topic guides focused on the interpersonal interaction between the patient and GP, the use of tools and resources during the consultation and interpreter use. Topic guides were revised throughout the data collection, informed by iterative data analysis. All transcripts were anonymized before beginning analysis.

### 2.6 Data analysis

Debriefing sessions between PP and the bilingual researchers were held after each interview to discuss and triangulate key findings, refine the lines of inquiry and identify the saturation of themes. Detailed meeting notes were taken, incorporating reflexive notes and contextual information to support the transparent development of emerging analysis. The interview data were analysed using Thematic Analysis as a method for identifying, analysing and reporting patterns (themes) within data,^32^ with data managed in Word and Excel. The first stage of analysis was familiarisation through debriefing sessions (P. P., H. N., N. J., A. H.) and intensive reading of the transcripts (P. P., S. B., D. M. M., L. T.). The second stage involved open and then focused coding, in which both inductive (data‐driven) and deductive (researcher‐driven) codes were identified across a subsample of the transcripts and then organized into a coding framework. This framework was then checked, further refined and applied to all the transcripts. The coded data were analysed and organized into different categories (i.e., language group, age, gender) to identify if there were any differences based on the sample characteristics and by data set. In discussion P. P., S. B., D. M. M. and L. T. identified relationships among and between categories to generate themes that formed the key findings. All final themes were also discussed and checked back with the other authors (D. Z., H. N., N. J., A. H.).

## 3 RESULTS

### 3.1 Participant characteristics

A total of 22 community members took part in the community participant interviews; eight were conducted in Arabic, six were conducted in Dari and eight were conducted in Tamil. Of the participants, 15 were female and 7 were male. The participants' time living in Australia ranged from less than 1 year to more than 10 years. Only one participant was a current asylum seeker and did not have access to Medicare. Six participants reported visiting their GP on a weekly basis, seven visited monthly and nine participants saw their GP less than four times a year. The community participant characteristics are outlined in Table 1.

**Table 1:** Community participant characteristics

Community participant characteristics	Number (%)
Sex	
Female	15 (68)
Male	7 (32)
Age	
18–29	6 (27)
30–39	4 (18)
40–49	5 (23)
50–59	3 (14)
60+	4 (18)
Country of origin	
Afghanistan	4 (18)
Iraq	4 (18)
Lebanon	1 (5)
Pakistan	2 (9)
Sri Lanka	8 (36)
Syria	3 (14)
Language other than English spoken	
Arabic	8 (36)
Dari	6 (27)
Tamil	8 (36)
Years living in Australia	
Less than 5 years	9 (41)
5–9 years	6 (27)
More than 10 years	7 (32)

A total of nine bilingual GPs working in Western Sydney took part in the provider interviews; of these participants, three were males and six were females. The GPs had, on average, worked for 17 years in general practice. However, this ranged from being recently qualified and so working for less than a year, to extensive experience across many decades.

We identified four themes reflecting both the community and GP participant perspectives: (1) A sense of broad ‘kinship’ with language concordant GPs; (2) divergences and dynamism in markers of PCC; (3) unaddressed misalignment when expectations are not verbalized and (4) unusual explicit description of expectations and healthcare norms. Integral to our analysis is the comparison of the findings from the community participant and GP participant datasets. As part of this process of triangulation, we have included some of the interpretations of the dual findings within the Results section.

### 3.2 Kinship with language concordant GPs

All the community participants interviewed reported that they deliberately chose to see a GP who spoke the same language as them. Removing the language barrier allowed them to easily converse with their GP.

*My problem is… it's easier for me to speak in my mother tongue. It is harder for me to explain things in another language*. (Tamil CM, P6)

Participants felt that GPs who spoke the same language were more approachable as they felt a presumed kinship to them based on their cultural and linguistic background. Many commented they felt that they were speaking to a trusted family member or friend.

*Yes, whatever problem we have, we tell her as if she is our sibling. She also gives us good advice*. (Tamil CM, P1)

### 3.3 Divergence and dynamism in markers of PCC

Although most participants felt linguistically understood by their GP and comfortable discussing their concerns, they sometimes still expressed dissatisfaction in the outcomes of their care. The presence of both invisible and visible actions was seen as tangible markers of care from the community members' perspective.

### 3.4 Invisible actions

Invisible actions generally revolved around the presumed personal and professional characteristics of the GP, as observed by the community participant. Personal characteristics, which were seen as beneficial by the community participants, were race, gender and language spoken. These unintentional visual cues and profiling of the GP by the community member allowed them to feel innate kinship with their GP and gave them a sense that they would have a shared cultural understanding with a GP who may look similar, but certainly sound like them. The perception is that, with the obvious language barrier removed, they are coming from the same cultural understanding and expectations of markers of good care.

*The face is exactly the same as Arabic face you can see the features is quite Arabic. And when they see an Arabic face, they find it more friendly, or they start more open on talk about it more openly. So, they feel more confident, more comfortable*. (Male GP, P7)

Professional characteristics of GP, which were highly regarded by the community members, were skills including empathy, active listening and mannerisms when engaging with patients. These taught skills, in combination with the GPs personal characteristics of language and cultural similarities, enabled the patients to feel comfortable and trust their GP. These skills are also considered core elements of PCC. These key skills involve creating a healing relationship, exchanging information, responding to emotions, and tailoring the care to the individual's clinical needs, life circumstances and personal preferences.

*They treat us in a nice way. They are humane, they have empathy*. (Arabic CM, P5)

*They give me enough time and I can talk about my problems. I can talk with ease until I finish*. [regarding Dari speaking GP] (Dari CM, P5)

### 3.5 Visible actions

Contrasting the invisible markers of care and cues from the GPs' presumed personal and professional characteristics, other markers of care were visible. Some of the community participants saw visible actions as positive markers of care; these were actions such as increased routine testing and physical examinations. Specifically, this involved things, such as blood tests, conducting blood pressure checks and sending the patient for imaging. Additionally, the visible action of sending for referrals to specialists and any further testing was also viewed as a positive action. Community participants reported that these visible actions that their GPs were doing allowed them to feel cared for.

*I found they care a lot. They took my blood pressure, blood tests, everything was done. They plan everything*. (Arabic CM, P3)

This suggests that community members identified and correlated visible actions with markers of good care from their GP. While most of the community participants commented that their GP took the time to listen to them and that shared language made them approachable, participants were dissatisfied in instances where the GP listened and gave no immediate resolution. For many this raised concerns that there was too much emphasis on listening and inadequate evidence of intervention, with tests, referrals and the arrangement of follow‐up visits being interpreted as delaying or postponing action. They often compared this to experiences in their home country where they would be seen by a doctor and would be given a diagnosis and treatment during the same consult, which to them had been a reassuring indicator there would be a rapid solution.

*I can go many times to the family doctor, and he will only listen to me. No more no less*. (Arabic CM, P1)

*There are problems like that here but over there we can just get things done straightaway. Over there, if we are unwell, we can just pay the fee and get the service*. (Tamil CM, P3)

### 3.6 Unaddressed misalignment when expectations are not verbalized

These expectations of visible actions highlighted an underlying fracture between community members' expectations and the care that they received, which they described in the interviews as ‘worrisome’. The community participants had not articulated this disconnect in their consultations with their GPs, nor discussed how their expectations had been shaped by their prior experience of care. This pattern was evident regardless of how long they had been living in Australia and did not differ by gender or age. Community participants described how they did not feel the need to voice their expectations; considering them normative and assuming that these norms were shared and that they should be addressed accordingly. This was commonly justified by the assumption that as the GP spoke the same language, this need not be said as they presumed that they would therefore have similar expectations too.

In the absence of some visible markers of care and explanation, there was dissatisfaction from the community participant perspective. Community participant narratives suggest that they expect to have all their healthcare needs resolved during a single visit. If those healthcare needs were not immediately met through direct action, interpreted as a proxy for resolution, they were viewed as indicative of a product of a slow system or that the GP was somehow delaying the inevitable progress of treatment or resolution.

*Here they have developed equipment, but they procrastinate. In general, equipment is better here but they take long to get to the diagnosis*. (Arabic CM, P3)

Contrastingly from the GP perspective, not providing immediate and perhaps unnecessary intervention or action, but rather investigating through justifiable and evidence‐based tests and follow‐up would be seen as the norm for providing PCC, as they are following what would be best practice in an Australian healthcare setting. This is concealed through a presumption that this was a normative decision‐making pathway to deliver PCC and, following a similar pattern to the community participants, ‘went without saying’. The disconnect between anticipated approaches of high‐quality care demonstrates how expectations based on cultural norms and expectations from different healthcare systems can create a point of misalignment.

*Medically speaking, it takes a lot of effort to cancel some of the medical stuff that they're told there, for example, I need treat cough with an antibiotic, for example. Simple as that*. (Male GP, P1)

This misalignment may be masked by the implicit emphasis within language concordance that presumes a shared understanding. Unfortunately, in the absence of community participants verbalizing their expectations of physical action to their GP, and the GP not articulating why action is not taken, there is a disconnect in the care they are given. These disconnects in the expectations and actions are not identified by the GPs, rather GPs felt that they were coming in from the same level of understanding. This created a mutual and perpetual misunderstanding of the expectations from both the community members and GPs. GP participants felt that having the same language and cultural backgrounds meant that there was an alignment of the understanding between them and their patients. In the example below, the GP felt that due to their language background they felt they knew what their Tamil‐speaking patients expected and needed.

*Our background like, you know, so if you are a Tamil speaking like doctor, so, you know that what is the background of, you know what it is they expecting, what they really need and that kind of thing, so that actually helped us*. (Female GP, P8)

The absence of action or some sort of physical intervention, which are markers of care based on previous experiences of healthcare, impede meeting the threshold of PCC from the community members' perspective. Suggesting that there is a misalignment in what constitutes PCC from the perspective of the GP who may speak the same language but is influenced by the Australian healthcare system. In this instance, PCC is limited, as it is not attending to the person's needs, even if this is to support them to adjust their expectations.

### 3.7 Unusual explicit description of expectations and healthcare system norms

Articulating expectations provide an opportunity for them to be addressed and recalibrated. Although unusual within our sample, some GP participants reported that they often acknowledge that their patients may be coming from a different healthcare system where they may have a ‘singular’ interaction with immediate care. This is contrasted to the Australian system where the GP builds an ‘ongoing’ interaction and takes into consideration the patients' needs and preferences in the process.

*Here that the patient is invited to be part of the management process. Whereas there, it's you either do A or you B or That's it. That's no other choice. So, it takes a bit of getting used to for a lot of them. But once they realize that, you know, ultimately we are actually after their own well‐being they understand really well*. (Female GP, P2)

Other strategies GPs employed to address patients' concerns were to invest time in explaining how the Australian healthcare system works and often guide them as they learnt to navigate the new system.

*I think its most important to explain to them how the Australian system runs. Because they don't have the knowledge. So, this misconception is there*. (Female GP, P6)

GPs' guidance was not in response to patients' articulation of misaligned expectations, but a few of them were able to address these unvoiced concerns indirectly by explaining the normative approaches adopted in the Australian healthcare system. These explanations from the GP were done in an effort to help their patients understand their reasoning. GP participants felt this additional information was appreciated by their patients to help them not only further engage with the Australian healthcare system but also recalibrate their understanding of the system. Highlighting those explanations resulted in an unconscious recalibration of the community member's expectations.

## 4 DISCUSSION

**Figure 1:**
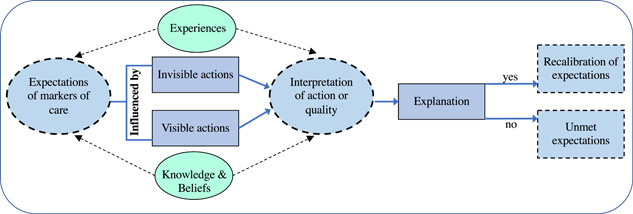
Model representing how community member expectations evolve depending on the presence of actions and explanations

The findings of the qualitative study illustrate that refugee community participants' expectations are formed by both their impressions of visible and invisible care or actions and previous experiences and encounters with healthcare systems. Community participants often interpret shared language as a proxy indicator of shared expectations. When their expectations are not met and the reasons for this disconnect are not made visible through explanation, then a misalignment persists, which shapes their interpretation that they are not receiving high‐quality PCC. If explanations were deliberately provided by GPs, anticipating a divergence in expectation shaped by prior exposure to alternative health systems, as part of early engagement in care (and potentially routinely revisited) this would allow for the recalibration of expectations that are more in line with, or at least more aware of, the norms of the current healthcare system (Figure 1).

Highlighted through participants' accounts, communication and quality of care are influenced by their expectations around the markers of PCC. Much of these expectations and subsequent interpretations of primary healthcare are formed through their experience and knowledge of other healthcare services. Interrogating the pertinence of shared language has illustrated how unreliable it is as a certain marker of shared experience or aligned expectations. Our findings emphasize the importance of applying the PCC approach to refugee and asylum seekers by discussing expectations and providing explanations for care pathways and the actions taken (or not taken). Recommendation for practice and research in Table 2.

**Table 2:** Suggestions for practice and future research

Suggestions for research
Promote and encourage explicit explanations of how prior experiences have framed expectations of good care to be able to provide PCC
Endorse healthcare provider explanation of the health systems and best practice
Record the impact of verbalizing the expectations of good care, in language‐concordant healthcare settings
Generate data to identify the cause of more frequent consultations and whether expectations change over the course of multiple visits

Abbreviation: PCC, patient‐centred care.

The complex influence of ‘unsaid’ expectations on evaluations of quality care has received inadequate attention to date. Community participants generally acknowledged that the reason they saw a language concordant GP was so that they could easily communicate and that they felt that someone from a similar ethnic background would understand them. Language alone can be an insurmountable barrier that makes navigating other social determinants of health, such as access to safe housing, employment and transportation—nearly impossible.^18^ Providing language‐concordant care is an opportunity for GPs and patients to collaboratively identify and plan to overcome some of these social factors, which impact their health and well‐being.^33^

Guided by the bilingual educators within the research team, we learnt that it is a common strategy for members from this community to deliberately seek out GPs who speak their language. As GPs were reported as approachable and trustworthy by all participants, they would remain the best source to provide not only accurate guidance and information but also provide clarification and explanation to help individuals realign what is not only a successful consultation but what are markers of good PCC.

Another explanation for the cause of the disconnect in the expectations of care could be that perhaps many refugees and asylum seekers presented to healthcare services in their country of origin with an acute problem. In this instance, there would be some sort of resolution to the acute and often symptomatic problem and this could be the norm in their country of origin.^34^ However in comparison in the Australian primary healthcare setting, there is a greater preventative healthcare lens that accounts for the routine testing and monitoring.^3^ There is not only a contrast in the healthcare systems but there could also be a contrast in the condition in which the patient presents themselves.

This study has some limitations. The generalisability of our findings is limited as the study was conducted in a specific location where community members were able to find and access language‐concordant GPs. Further, only one participant was an asylum seeker. It is likely that their precarious access to state‐supported healthcare through the Medicare system frames the specificity of their experience and warrants further investigation. A strength of our study was that in each stage of the study efforts were made to make engagement more inclusive by providing explanations in participants' preferred language. This was achieved through the provision of accessible information through the video information sheet, as well as partnering with the WSLHD multicultural health team who have already trusted actors within the community, which facilitate access, reach and rapport. An additional strength was that by purposive sampling for different language groups we were able to engage participants from six different countries, allowing for different perceptions of expectation of primary care.

## 5 CONCLUSION

This study highlights the importance of understanding that language concordant care does not always equate to uniform expectations around the markers of care. By making explicit the points of disconnect, between refugee and asylum seeker community members and GPs, our study illuminates how these could be addressed, such as by verbalizing those unsaid expectations. This could be done by asking the community member what would be seen as good PCC from their perspective, what their prior expectations of good quality care are and what they see as markers of a successful consultation. Future interventions for general practice need to consider the benefits of addressing expectations, with consideration on how it could be useful within the available consultation time. By refugee and asylum seekers articulating their expectations, GPs are given the opportunity to address those expectations through traditional avenues of PCC, such as listening, consideration of social circumstances and addressing the needs and preferences of the individual.

## CONFLICT OF INTERESTS

The authors declare that there are no conflict of interests.

## AUTHOR CONTRIBUTIONS

Pinika Patel, Danielle M. Muscat, Lyndal Trevena, Dipti Zachariah and Sarah Bernays conceived this study and its design. Pinika Patel, Hanaa Nosir, Nishanthie Jesurasa and Amina Hadi conducted the data collection. Pinika Patel and Sarah Bernays conducted the coding and analysis. All authors were involved in interpreting and discussing the results. All authors commented on and approved the manuscript.

## Data Availability

The data that support the findings of this study are available from the corresponding author upon reasonable request.
